# The Association Between MIND Diet Adherence, Nutritional Status, and Psychosomatic Health in Adults Aged 60+: A Pilot Study

**DOI:** 10.3390/healthcare14050598

**Published:** 2026-02-27

**Authors:** Bogusław Stelcer, Magdalena Czlapka-Matyasik, Małgorzata Woźniewicz, Maria João Campos, Jacek Anioła

**Affiliations:** 1 Department of Human Nutrition and Dietetics, Faculty of Food Science and Nutrition, Poznań University of Life Sciences, Wojska Polskiego 31, 60-624 Poznan, Poland; boguslaw.stelcer@up.poznan.pl (B.S.); malgorzata.wozniewicz@up.poznan.pl (M.W.); jacek.aniola@up.poznan.pl (J.A.); 2 LAQV, REQUIMTE, Laboratory of Bromatology, Pharmacognosy and Analytical Sciences, Faculty of Pharmacy, University of Coimbra, 3000-548 Coimbra, Portugal; mcampos@ff.uc.pt

**Keywords:** nutrition, diet quality scores, health, well-being, adults

## Abstract

**Highlights:**

**What are the main findings?**

- Nutritional status (MNA) is a primary determinant of mental health, with malnutrition risk significantly increasing levels of distress, anxiety, and depression in seniors.
- Consumption of unhealthy foods (nHDI) is directly linked to psychological symptoms, whereas strict adherence to the MIND diet showed no significant correlation in this group.

**What are the implications of the main findings?**

- Geriatric interventions must integrate routine nutritional screening with psychological assessment to identify vulnerable individuals and support resilient ageing.
- Public health policies should prioritize reducing unhealthy food intake and improving overall nutritional status as key strategies for protecting senior mental health.

**Abstract:**

**Background/Objectives**: Ageing is associated with reduced adaptive capacity, which may influence responses to chronic stress and contribute to adverse lifestyle changes. This study examined the relationships among diet quality, nutritional status, and psychosomatic health in adults aged 60+, while considering the role of psychological stress. **Methods**: A total of 372 participants were assessed using a validated FFQ to evaluate diet quality, the MNA to determine nutritional status, and anthropometric measurements. Psychological functioning was measured using the PSS-10, the 4DSQ, and the geriatric GDS scale. **Results**: No association was identified between adherence to the MIND diet and depressive or anxiety symptoms. However, depressive symptoms were positively associated with the consumption of unhealthy foods. Gender differences also emerged: women reported higher levels of perceived stress (PSS10 F: 13.5 M: 10.5; *p* < 0.001), anxiety (F: 0.97; M: 0.39; *p* < 0.01), and somatisation (F: 6.18; M: 4.22; *p* < 0.001), suggesting greater vulnerability to everyday stressors. Participants at risk of malnutrition displayed significantly higher levels (*p* < 0.05) of stress (8.33), depression (0.73), and anxiety (1.76) compared with well-nourished individuals (5.03; 0.33; 0.77, respectively). **Conclusions**: These findings underscore the significant relationship between nutritional status and mental functioning in older adults. They emphasise the need to integrate nutritional assessment with somatic and psychological evaluation to better support the health and well-being of seniors and to improve understanding of the interactions between diet, stress, and psychosomatic functioning in the ageing process.

## 1. Introduction

Behavioural patterns have a key, scientifically proven impact on the mental health and well-being (self-esteem, well-being, cognitive function) of seniors. Nutrition is perceived as a health-related factor in psychology and medical science. In recent years, the number of studies examining the effects of nutrition on mental state has dramatically increased, resulting in a better understanding of the qualitative consequences and side effects related to mental health. It has been established that nutritional supplements can help combat the effects of stress, improve mood, and reduce anxiety. There is an increasing number of studies on health-promoting behaviours in various areas of health among older adults that have a positive impact on prolonging physical fitness, mental well-being and independence, thereby contributing to successful ageing.

### 1.1. Defining Health Factors for Seniors

In Poland, the number of adults aged 65+ is expected to increase by almost 50% over the next 15 years. Such rapid demographic change is occurring in several countries worldwide, making this phenomenon one of the most significant social trends of the 21st century. In Poland, the life expectancy for men is 75.7 years and for women it is 81.9 years; both have been increasing in recent years [1]. However, the factors that for years have been most responsible for the decreasing years of life in the health of Poles are smoking, poor nutrition and high blood pressure [2]. The ageing process has social and economic consequences, placing a burden on the healthcare system. The cost of treating people aged 65+ is higher than that of treating those aged 15–54 [3]. As a result, medical procedures are being developed to enhance the health and quality of life for adults aged 60+. Among the most important in geriatric prevention are the detection and treatment of obesity and malnutrition. Adults aged 60+ are one of the most neglected segments of the population in terms of medical care, and malnutrition significantly increases the risk of morbidity and mortality [4,5]. Therefore, promoting optimal nutritional status, access to health resources, and engaging in physical and social activities are key to improving longevity and quality of life among adults aged 60+. Malnutrition in this group worsens overall functioning, reduces cognitive performance and predisposes individuals to age-related degenerative diseases [6]. Among the factors significantly affecting the nutritional status of adults aged 60+, pathophysiologic changes are a major contributor. In seniors, there is a decrease in fat-free mass (FFM), accompanied by a simultaneous increase in total body fat (TBF) [7]. Other factors affecting the nutritional status of seniors include socioeconomic problems (loneliness, poverty, lack of social support), psychological problems (depression, stress, having to cope with the hardships of daily life), disability, or lack of nutritional knowledge. Risk factors for lifestyle diseases are inappropriate health behaviour, improper dietary style, and overweight and obesity.

On the other hand, modifying seniors’ lifestyles (changing dietary patterns, incorporating physical activity, and developing coping mechanisms alongside the aforementioned factors) is considered an important preventive measure [8]. While the impact of healthy dietary patterns, such as the Mediterranean or DASH diets, on mental health has been extensively reviewed, there remains a need for further research focusing specifically on the MIND diet in the context of psychosomatic health in subjects aged 60+ [9]. Lalonde’s concept of health fields [10]. This concept identifies the determinants of health, including human biology, environment, lifestyle, and healthcare organisation. It emphasises that health is not determined solely by medical interventions but also by factors outside the traditional healthcare system, such as lifestyle, including dietary behaviour and psychosocial factors. According to Tulchinsky, the first International Conference on Health Promotion, organised by the WHO in Ottawa, established a formal definition of health promotion that became the conceptual impetus for the development of a new discipline and a significant factor in public health policy [10]. Since then, the Ottawa Charter has adopted the definition of health promotion as: “*the process of enabling people to increase control over, and to improve, their health. To achieve a state of complete physical, mental, and social well-being, an individual or group must be able to identify and realise their aspirations, satisfy their needs, and adapt to or change their environment. Therefore, health promotion is not just the responsibility of the health sector, but goes beyond healthy lifestyles to well-being*”. The emphasis, among other things, is on developing individual skills, as behaviour and lifestyle play a key role in promoting health.

Additionally, the WHO suggests that up to 50% of a person’s health is influenced by lifestyle factors, including a proper diet and regular physical activity [11]. Older people are often affected by multiple chronic diseases at the same time. These are classified as chronic non-infectious diseases, of which poor nutrition is an important risk factor, and often the primary treatment method is a proper diet. In addition, several research papers suggest that positive functioning across various dimensions of social relationships (e.g., structural, functional, qualitative) is also strongly associated with better health indicators [12,13,14]. Psychosocial resources are often included in the conceptualisation of groundbreaking gerontological and geriatric models that have emerged to characterise processes and outcomes that promote positive ageing in the face of increasing adversity in late adulthood. In this context, numerous research reports are emerging that include empirical work on: “successful ageing”, “optimal ageing”, “efficient ageing”, “resilient ageing” and “positive ageing” [15].

### 1.2. Well-Being and Resilience as a Goal for Healthy Ageing

Over the past 100 years, life expectancy has increased by almost 30 years [16,17]. The above situation necessitates the development of adaptation mechanisms to support the growing number of seniors, enabling them to function correctly and effectively within their social environment. Spheres requiring adaptation include social loss, deterioration in health, and the need to withdraw from existing social roles. However, in the face of these multifaceted and accumulating losses in old age, some people successfully cope with the challenges of senior age. The question remains: who are these individuals, and what social determinants of health and individual-level factors contribute to their resilience? What kind of coping mechanisms and lifestyle factors allow them to adapt in the face of increasing losses associated with late adulthood? Identifying factors that enhance health resources and resilience among those best able to adapt to the demands of old age is important for the formulation of dietary recommendations and health interventions.

Psychological well-being has been defined in various ways, and while its exact content and contours are contested and evolving with new empirical research and theoretical models [18], two main perspectives characterise its basic features. The first is the eudaimonic approach, which defines psychological well-being as a person’s ability to identify meaningful goals and pursue them through noble actions to achieve ultimate potential [19]. The second approach is the hedonistic approach, which defines psychological well-being as characterised by a high frequency of positive affect, a low frequency of negative affect, and life evaluations that are satisfying [20]. The well-being of seniors is a widely debated category and is most often understood as an individual’s sense of satisfaction, which relates to the personal resources an individual possesses. It is a category related to the eudaimonistic tradition, which is multidimensional [19,21]. It is associated with a multidimensional approach to improving quality of life, encompassing physical, mental, social and spiritual health. This includes maintaining an active social life, engaging in regular physical activity, following a healthy diet, and having access to family and social support [22]. An individual’s mental resilience comprises a set of diverse competencies, abilities, traits, resources, and processes related to the development and maintenance of healthy adaptation [23,24,25]. This issue is an attractive target for psychological research and intervention compared to alternative definitions of healthy ageing that often emphasise objective health [25]. Seniors can employ adaptive strategies, including eating behaviours, among others, to achieve a subjective sense of personal well-being and fulfilment [15]. Models of healthy ageing assume that there is no single agreed-upon set of criteria that older adults should strive for [26]. However, focusing on resilience enables individuals to identify their own adaptive response patterns, which reflect their personal values [27]. No single factor has an overarching effect on resilience [22]; rather, several antecedents contribute to it [22]. Developing one’s own resilience resources allows individuals to create adaptive response patterns that reflect their own values [27]. In this report, we focused on assessing lifestyle factors, particularly dietary patterns and the effects of stress, that impact health. As other research suggests, these factors are believed to contribute to healthy ageing [23]. In this report, we focused on assessing lifestyle factors, particularly dietary patterns and the effects of psychological stress, that contribute to health. A growing body of research suggests that these factors promote healthy ageing.

We hypothesised that there is a relationship among nutritional status, eating behaviour, and selected psychological functioning parameters. Of particular interest was the relationship between the nutritional status of the study sample and the severity of perceived stress, anxiety and depressive moods. The study aimed to determine the impact of nutrition on mental well-being and to identify nutritional factors that influence seniors’ well-being, along with other cognitive and somatic indicators.

## 2. Materials and Methods

### 2.1. Participants and Design

The study participants were selected voluntarily, without randomisation. Recruitment for the study took place among students of the third-age universities and members of local senior groups, as well as through advertisements in pharmacies and public health centres in the Wielkopolska Province. Each participant was informed of the study’s purpose and procedures, as well as the option to withdraw at any time. Informed consent to participate in the study was confirmed in writing by the study participant at any stage. The study adhered to the principles of Good Clinical Practice and met the ethical standards outlined in the Declaration of Helsinki [28]. The study protocol was approved by the independent Bioethics Committee of the Poznan University of Medical Sciences (957/19). The study included 372 subjects aged 60 years or older (Table 1). The study comprised 79% women and 21% men. Most of the study sample was socially active and had completed secondary or higher education (83%). The inclusion criterion was being over 60 years of age. The exclusion criteria were current or past oncological treatment within the last 5 years, the presence of severe conditions such as chronic kidney and liver disease, and untreated or uncompensated endocrine disorders, as both the disease and treatment significantly affect patients’ eating behaviours, as well as physical limitations preventing visits to the laboratory and cognitive limitations preventing communicative interaction. The entire set of questionnaires, tests, and measurements was conducted during a single visit to the laboratory by the subjects.

### 2.2. Measurement Tools

#### 2.2.1. Mini Nutritional Assessment (MNA)

The Mini Nutritional Assessment (MNA) screening tool comprises a 22-question questionnaire that evaluates the nutritional status of elderly patients through anthropometric measurements and dietary and subjective assessments. The total score of patients was categorised as well-nourished (MNA ≥ 24), at risk of malnutrition (MNA 17–23.5), or malnourished (MNA < 17). The MNA is validated and widely used due to its reliability and ease of use (scale sensitivity: 97.9%; scale specificity: 100%) [29].

#### 2.2.2. Food Frequency Questionnaire (FFQ)

This questionnaire assesses the frequency of consumption of selected food products and was used to determine the subjects’ dietary patterns using the a posteriori method. The method utilises nutritional quality indicators, including adherence to the Mediterranean diet and diet quality indexes, as described below. The questionnaire used had previously been validated in the SENECA study [30]. The questionnaire covered commonly consumed food products among the Polish population and consisted of 57 items, including individual foods and various food groups. Respondents were required to specify how often they had consumed the listed products during the month preceding the study, both as single items and as ingredients in dishes. The frequency of consumption was classified according to the following scale: 4–5 times a day, 2–3 times a day, once a day, 4–6 times a week, 2–3 times a week, once a week, 2–3 times a month, once a month, less often or never [31]. The consumption of products from the qualitative data was then transformed into numerical data to obtain results in the form of monthly frequencies: 4–5 times a day (135 times/month), 2–3 times a day (75 times/month), once a day (30 times/month), 4–6 times a week (20 times/month), 2–3 times a week (10 times/month), once a week (4 times/month), 2–3 times a month (2.5 times/month), once a month (1 time/month), less often or never (0 times/month).

Regarding dietary indexes, a literature review identified two [31,32,33,34,35,36]. (1) “Pro-Healthy Diet Index” (pHDI-8)—this index involved 8 food groups with a potentially beneficial influence on health, as sources of high-quality proteins, low glycemic index, fibre, symbiotic abilities, and sources of vitamins and minerals. (2) “Non-Healthy Diet Index” (nHDI-11)—this index involves 11 food groups with a potentially negative influence on health, as simple sugars, high glycemic index, animal fats, saturated and trans fatty acids, cholesterol and alcohol. In order to calculate healthy and non-healthy dietary indexes (pHDI-8, nHDI-11) using the frequency of the food intake, all products were grouped into the two mentioned food categories by naming them as healthy (1—wholemeal bread, coarse grains, oat flakes and whole meal pasta; 2—milk, including flavoured milk; 3—fermented milk drinks, e.g., yoghurts, kefirs; 4—cottage cheese, including homogenised cheese, cottage cheese desserts; 5—white meat dishes, e.g., chicken, turkey, rabbit; fish; 6—dishes made from legumes, e.g., beans, peas, soybeans, lentils; 7—fruit; 8—vegetables) or non-healthy products (1—white bread; 2—white rice, plain pasta or small grains; 3—fast food; 4—butter and lard as an addition to bread or dishes; 5—yellow cheese; 6—cold cuts, sausages or frankfurters; 7—dishes made from red meat; 8—sweets; 9—sweetened carbonated or non-carbonated drinks; 10—energy drinks; 11—alcoholic beverages). The product consumption frequencies were grouped into two categories and expressed as daily consumption per person, in accordance with the guidelines [32]. Both total scores were calculated according to the procedures described in previous studies and then analysed into tertiles [33,34,35,36,37]. The pHDI-8 index measured the consumption intensity of 8 healthy products, while the nHDI-11 index measured the consumption intensity of 11 non-healthy products. Frequencies of intake for each food component, healthy or non-healthy, were separately summed to obtain a total pHDI-8 index ranging from 0 to 16 and a total nHDI ranging from 0 to 22. The interpretation of the pHDI and nHDI was intuitive. The higher values indicated a higher intensity of consumption for the foods listed in the index.

Based on data from the FFQ questionnaire, the degree of adherence to the MIND diet (Mediterranean-DASH Intervention for Neurodegenerative Delay) was also calculated and expressed in points. The MIND diet contains recommendations for 15 food components, including 10 food components considered healthy for the brain (i.e., green leafy vegetables, other vegetables, nuts, berries, beans, whole grains, fish, poultry, olive oil, and wine) and five unhealthy food components (i.e., red meat, butter and stick margarine, regular cheese, fried and fast foods, and cakes and sweets) [38]. For each food component listed, a score of 0 was assigned if participants did not follow the recommendations, 0.5 for moderate adherence, and 1 for good adherence, expressed as the number of standard servings of a given product per week. Wine was excluded from the products considered healthy in this study, as recent reports indicate that there is no safe level of alcohol consumption, especially in seniors, in whom its intake increases the risk of various diseases, as well as other health-related issues [39]. The scores for each food component were summed to obtain a total score ranging from 0 to 14. Values 12.5–15 indicated high adherence, 8.5–12 moderate adherence, <8 poor adherence to the MIND.

#### 2.2.3. The Four-Dimensional Symptom Questionnaire (4DSQ)

The 4DSQ questionnaire is a 50-item self-report validated inventory consisting of four scales measuring 16 items [40]. The distress scale measures a subject’s most general and basic response to stress, whether from work or family demands, psychological difficulties, or life events. The Polish version displayed satisfactory psychometric quality in terms of reliability, internal structure and validity. Reliability coefficients (Cronbach’s alpha) were above 0.8 [41]. The depression and anxiety scales identify specific symptoms of depressive and anxiety disorders that are severe enough to warrant treatment. The somatisation scale measures symptoms that are associated with somatic stress. Responses are given on a 5-point frequency scale from “no” to “very often or constantly.” To calculate total scores, responses are coded on a 3-point scale: “no” (0 points), “sometimes” (1 point), “regularly” (2 points), “often” (3 points), and “very often or constantly” (4 points). By combining the response categories of “regularly,” “often”, and “very often or constantly,” relatively more weight is given to the number of symptoms experienced than to their perceived frequency.

The 4DSQ is a convenient tool for assessing the prevalence of psychosocial complaints [42]. The questionnaire detects almost all cases of subjects who suffer psychologically, regardless of the specific cause. In addition, it detects patients who are at relatively high risk of developing major depressive or anxiety disorders. The 4DSQ questionnaire is available free of charge for non-commercial use at www.4dsq.eu. Psychological disorders in this study were defined as present if patients scored in the medium- or high-risk range on one of the 4DSQ domains [40,42,43].

#### 2.2.4. Geriatric Depression Rating Scale (GDS)

The GDS is the scale used for screening for depressive conditions in elderly individuals. The 15-item abbreviated version, developed by Yesavage, was used in the study. The Cronbach’s alpha reliability coefficient was α = 0.94, identical to the split-half reliability of this instrument (r = 0.94). The sensitivity and specificity of the GDS were 84% and 95%, respectively [44]. Participants are asked to answer “yes” or “no” regarding how they have felt over the past few weeks. The scores for assessing depression were as follows: 0–5 points—no depression; 6–10—moderate depression; 11–15—severe depression [45].

#### 2.2.5. Perceived Stress Scale (PSS)

This questionnaire is a commonly used psychological tool to measure perceived stress. It is a measure of the degree to which the subject rates life situations as stressful. The questionnaire’s items assess the extent to which respondents’ lives are unpredictable, uncontrollable and overloaded. The scale also includes a series of direct questions about the current level of stress experienced. The questions are general in nature and therefore relatively free of content specific to any subpopulation. Questions in the PSS relate to feelings and thoughts over the past month [46]. The PSS scale questionnaire consists of 10 questions that assess subjective feelings about stress caused by personal problems and events, as well as effective coping strategies. Each question was rated on a Likert scale, where 0 = never; 1 = almost never; 2 = sometimes; 3 = quite often; and 4 = very often. Raw scores of 0–13 are considered low, 14–26 moderate, and 27–40 high.

### 2.3. Statistical Analysis

After considering the confidence level (90%) and the margin of error (5%), the calculated minimum sample size was 273 subjects [47]. Statistica v. 13.3 statistical software was used to calculate the sample size and study power [48].

Continuous variables were presented as means and standard deviations (SDs), and variables from scaling were presented as medians and percentiles (33.3% to 66.6%). The normality of continuous variable distributions was assessed using the Shapiro–Wilk test. The MNA tool was validated by calculating Spearman’s rank correlation coefficients between the overall MNA score and each of the 18 MNA items. The correlation coefficients between selected variables were calculated using Pearson’s or Spearman’s correlation, depending on the data’s normality.

The logistic regression analysis examined significant associations among adherence to the MIND and the severity of depression (GDS), level of perceived stress (PSS10), scores on four dimensions of mental health (4DSQ), and malnutrition risk (MNA). The dependent variables were the three levels of adherence to the MIND diet, while the independent variables were the selected psychological variables and risk of malnutrition.

An alpha of <0.05 was considered statistically significant. Data were analysed using STATISTICA 13.3 software (StatSoft Inc., Tulsa, OK, USA).

## 3. Results

### 3.1. Correlation Coefficients of Nutritional Status, Dietary Patterns, Psychological Symptoms and Other Parameters

The results presented in Table 2 highlight key relationships between psychological parameters and selected variables, especially non-healthy and healthy eating behaviours. Despite the low linear correlation coefficients, some were found to be statistically significant.

The age of the study participants was positively correlated with the severity of depression and somatisation, defined as the physical experience of stress and mental health problems. Body mass (BMI) was also directly related to somatisation, while the number of cigarettes smoked daily was directly related to the severity of anxiety and restlessness.

The intensity of depression correlated with the intensity of intake of non-healthy foods (nHDI) (*p* = 0.023). There was no relationship between the psychological parameters and adherence to the MIND diet. The respondents’ age correlated positively with 4DSQ—depression (*p* = 0.016) and 4DSQ—somatisation (*p* = 0.016), while BMI correlated with 4DSQ—somatisation (*p* = 0.013). There was also a positive correlation between the number of cigarettes smoked per day and 4DSQ—anxiety values (*p* = 0.010).

### 3.2. Psychological Symptoms and Nutritional Status in Relation to the Gender of the Study Sample

Statistically significant differences between genders are shown in Table 3.

Analysis revealed that women presented higher values of distress, anxiety and somatisation indicators. The results clearly revealed a higher level of overwhelm among the women due to daily events. The mental and physical health of the studied women was under greater pressure. It was shown to have a more substantial impact on various aspects of life, including psychosomatic symptoms, depression and anxiety. The PSS-10 results indicate that the women and men in the study sample do not rate their living situation as particularly stressful, uncontrollable or aggravating. The results measured by the Geriatric Depression Scale (GDS) are similar, indicating the absence of depressive symptoms in both genders. Nevertheless, women had significantly higher GDS values than men.

### 3.3. Psychological Symptoms and Nutritional Status in Relation to the Non-Healthy Diet Index

A comparison of psychological parameters and nutritional status in the study sample, in relation to different levels of the non-healthy diet index (nHDI), is presented in Table 4.

Statistically significant differences were observed in levels of depression (4DSQ and GDS) and anxiety (4DSQ) across increasing tertiles of the nHDI. The increase in the severity of depression and anxiety was associated with more frequent consumption of products considered unfavourable to health. However, no such relationships were observed for nutritional status (MNA) and perceived stress (PSS-10).

### 3.4. Psychological Symptoms in Relation to the Nutritional Status in the Study Sample

Table 5 presents differences in psychological parameter levels by degree of malnutrition, as assessed by the MNA score. The results showed that none of the subjects in the study sample had a value indicating malnutrition. However, in 33 subjects (8.87%) of the study sample, an MNA score indicated a risk of malnutrition. Moreover, statistical analyses revealed a significant effect of nutritional status level (well vs. risk of malnutrition) on all psychological symptoms in the studied group.

In the group of subjects with a risk of malnutrition, significantly higher measures of distress, depression, anxiety, and somatisation were revealed. For subjects classified as at risk of malnutrition, the magnitudes of adverse psychological health indicators (stress, depression, anxiety) were found to be statistically significantly higher than those of the others (well nourished).

Table 6 presents the associations between adherence to the MIND diet and selected mental health parameters and nutritional risk. Depressive symptoms, like mild or severe depression according to the GDS, increased more than two-fold with low adherence to the MIND diet (Adjusted OR = 2.13; 95% CI: 1.00–4.56; *p* = 0.050). Conversely, moderate or high perceived stress (PSS10) was associated with a 40% lower likelihood of high adherence to the MIND diet (Adjusted OR = 0.60; 95% CI: 0.38–0.93; *p* = 0.023).

Finally, logistic regression revealed that participants with middle or high adherence to the MIND diet had a significantly lower risk of malnutrition (Adjusted OR = 0.37; 95% CI: 0.16–0.85; *p* = 0.019), whereas low adherence was associated with a nearly three-fold higher risk of malnutrition (Adjusted OR = 2.70; 95% CI: 1.17–6.19; *p* = 0.019).

## 4. Discussion

The research presented here fits within the broader context of interdisciplinary work that combines clinical, health, and psychiatric psychology with the science of human nutrition and dietary patterns. In recent years, there has been an unprecedented increase in interest in the importance of diet in regulating mental well-being and overall health, particularly in the context of psychiatric care [46,49]. Nutritional psychiatry and psychodietetics are new, rapidly developing multidisciplinary fields. Over the past decade, there has been substantial growth in research on the role of dietary interventions in psychiatry and psychotherapy [50,51]. Relevant cross-sectional and longitudinal studies have shown that the more Western or highly processed foods a person consumes, the more likely they are to experience mental symptoms such as depression and anxiety [52,53,54,55].

On the other hand, studies show that the more the population follows a Mediterranean diet or other healthy eating habits, such as consuming more vegetables and fruits, the more they are protected against the development of mental disorders [51,56,57]. Several studies have shown that diet precedes the onset of psychiatric symptoms. Depressive disorders, due to their chronic and recurrent nature, remain one of the most significant challenges of modern medicine and dietetics [58]. One of the factors that increases the risk of psychological health disorders, such as depressive disorders, is diet. This trend is confirmed by our study’s results, which show deterioration in mental functioning among individuals at risk of malnutrition, as well as those with poor diet quality, reflected in the high intensity of unhealthy food consumption. Our study reveals the connections among psychological stress intensity, diet, and mental well-being.

In this study, we used mental well-being indicators seen through the prism of a correlation between the age of seniors and the severity of depression and somatisation, as measured using the 4DSQ questionnaire. A positive correlation was also demonstrated between BMI and the occurrence of somatic complaints. The study highlights, as noted in the literature, that older individuals who have lived longer are more likely to experience somatic complaints associated with ageing [59,60,61]. Furthermore, the severity of somatic symptoms increases with increasing body mass index (BMI). Also, a correlation was found between the number of cigarettes smoked per day and the severity of anxiety. So, anxiety may be interpreted as a risk factor for tobacco use, which has already been emphasised many times in the literature [62,63,64].

The present study’s classification of food products into ‘healthy’ and ‘non-healthy’ categories aligns with established dietary patterns such as the MIND (Mediterranean-DASH Intervention for Neurodegenerative Delay) and Healthy Low-Carbohydrate diets. Our findings, which associate nutrient-dense intake with better mental health outcomes, are consistent with recent research by Arabpour and Milajerdi (2025) and Barkhordari et al. (2022), who demonstrated that high adherence to the MIND diet significantly reduces the prevalence of anxiety and psychological distress [65,66]. However, we acknowledge that the health impact of these foods is context dependent. While energy-dense, ‘non-healthy’ products may be health-promoting in malnourished populations by providing essential calories, in our study population, their overconsumption is more likely linked to adverse outcomes. Furthermore, as Çakır and Özge (2025) suggest, the relationship between nutrition and mental state is likely bidirectional [67]. A ‘vicious cycle’ may exist in which heightened stress levels drive the consumption of refined carbohydrates and sweets—items categorised here as ‘non-healthy’—which may subsequently exacerbate the physiological stress response through adrenaline surges and blood sugar fluctuations. This bi-directionality, coupled with the potential influence of socioeconomic status on food choice, suggests that our observed correlations should be interpreted as part of a complex, reciprocal system rather than a simple linear cause.

Among the indicators of diet quality assessed, only the non-healthy diet index was associated with depression, suggesting a connection between the two. Multivariable logistic regression analysis (Table 6) revealed that high adherence to the MIND diet is a significant protective factor against perceived stress (OR = 0.60, 95% CI: 0.38–0.93). These results align with those of Barkhordari et al. (2022), who found that the MIND diet’s rich antioxidant profile may mitigate the physiological effects of psychological stress [65]. Interestingly, our study also highlighted a critical vulnerability in the low-adherence group (1st tertile), where the risk of mild or severe depression was more than doubled (OR = 2.13, 95% CI: 1.00–4.56). This correlation corroborates the research by Ebrahimpour-Koujan et al. (2019), emphasising that it is not merely the restriction of specific macronutrients but the high-quality of food sources—typical of the MIND pattern—that determines mental health outcomes [68].

Furthermore, the most striking finding in our analysis was the nearly three-fold increase in the risk of malnutrition among participants with low adherence to the MIND diet (Adjusted OR = 2.70, 95% CI: 1.17–6.19). This supports the argument that ‘non-healthy’ or energy-dense dietary patterns often fail to provide essential micro- and macronutrients, leading to subclinical deficiencies that may further impair emotional regulation. As noted by Çakır and Özge (2025), this can create a self-perpetuating cycle in which poor nutritional status diminishes mental resilience, which, in turn, leads to further poor dietary choices [67]. Our results suggest that the MIND diet may act as a dual-purpose intervention, simultaneously addressing nutritional deficits and psychological well-being.

However, our research is consistent with the existing literature on this subject; it is challenging to clearly determine whether poor dietary habits and their consequences, such as malnutrition, contribute to depression, or whether depressive states may influence diet and, consequently, the nutritional status of seniors [69,70]. We can undeniably conclude that dietary patterns, particularly unhealthy eating behaviours among our respondents, remain closely linked to their mental health and well-being, as confirmed by the extensive literature and demonstrated in our previous studies [37,71,72,73,74]. It is worth emphasising that, as described in the literature and possibly observed in our studies, the correlations may also reflect a ‘vicious cycle’ in which poor mental states, such as high stress or low mood, trigger compensatory cravings for energy-dense foods, which, in turn, may further destabilise emotional well-being through physiological mechanisms [67].

It was noted that anxiety and depression were more serious factors accompanying unhealthy eating habits in the study population. This study confirmed the relationship between nutritional status and mental functioning in seniors aged 60+. Individuals at risk of malnutrition showed a statistically significant increase in mental symptoms and perceived stress. This phenomenon is of interest to psychology and the health sciences [75]. With the right lifestyle and eating habits, seniors’ health and well-being can be improved and strengthened. Late adulthood can be experienced from the perspective of ‘positive ageing,’ for which health-promoting behaviours are essential. The health behaviours of older adults vary widely in association with sociodemographic characteristics [76].

The consequences of a poor diet affect other global health threats. Alzheimer’s disease and depression are comorbidities of obesity, confirming concepts suggesting that cardiovascular disorders may play a role in the development of dementia and psychiatric pathologies [57]. In summary, nutritional strategies and interdisciplinary psychodietetic interventions appear to be an intriguing area of research that warrants further exploration [77]. A randomised controlled trial published in *BMC Medicine* demonstrated the beneficial effects of a three-month nutritional intervention on moderate and severe depression, with significantly greater improvement in the intervention group (remission was achieved in 32% of individuals) compared with the control group [78].

The current study revealed the role of gender, pointing to poorer mental functioning in women compared to men. Women experience stronger symptoms of stress, depression, and anxiety than men. The results obtained in this study indicate a higher level of overwhelm from everyday events among the women surveyed. The impact of stress on various aspects of life, including psychosomatic symptoms, depression, and anxiety, are more substantial in women than in men. The women surveyed likely experienced higher levels of anxiety in situations that were difficult to control. This can lead to long-term health problems, ranging from somatisation to negative consequences for mental health. Another possible explanation is that the women studied may have felt overwhelmed by too many responsibilities or difficulties beyond their coping abilities. Overload leads to increased stress levels, which can manifest themselves in psychosomatic symptoms such as headaches or sleep problems. The discussion on women’s health has been ongoing for years, and various recovery methods have been described. Among the solutions mentioned are a well-balanced diet, social support, physical activity, and other factors [58,79].

The study has certain limitations. The selection of participants was deliberate. Further analysis is needed to examine the lives of seniors across different social groups, especially those who differ in education, nutritional knowledge, place of residence, and economic status. Due to the cross-sectional design of this study, it is impossible to establish a definitive causal relationship, leaving open the possibility of reverse causality in which an individual’s mental health status dictates their dietary patterns rather than the other way around. Furthermore, socioeconomic status and educational attainment may act as significant confounding variables, as they simultaneously influence both access to health-related knowledge and the economic capacity to maintain specific dietary habits.

Another limitation of the study is the significant gender imbalance, with women accounting for 79% of participants. This disproportion is characteristic of the elderly population in Poland (the feminisation of old age) and reflects women’s greater willingness to participate in health-oriented research. However, it limits the generalisability of the results to the male population. Due to the small size of the male subgroup (*n* = 78), a separate statistical analysis for gender was not performed to avoid the risk of statistical bias and underpowered conclusions.

The variables analysed were assessed using a self-reporting technique. This means there is a risk of error, as respondents assessed their behaviour subjectively and the tendency to present oneself in a better light may be significant in assessing specific health behaviours. The study was cross-sectional, which does not allow for clear conclusions about cause-and-effect relationships. Despite the limitations presented, the study makes a valuable contribution to the field of nutrition and mental functioning in older adults.

## 5. Conclusions

Our study demonstrates that high dietary quality, specifically adherence to the MIND diet, is strongly associated with reduced stress, anxiety, and depression in adults aged 60+. Conversely, malnutrition risk and high processed food consumption (nHDI) significantly exacerbate psychological distress and somatic symptoms. Factors such as female gender, advanced age, and higher BMI further increase vulnerability. These findings highlight the need to integrate nutritional screening (MNA) into geriatric mental healthcare and to promote neuroprotective diets as a non-pharmacological intervention for healthy ageing.

## Figures and Tables

**Table 1:** Characteristics of the study sample.

Characteristics	Data
No. of individuals	372
No. of females (no./%)	295 (79)
At the age of 75 or older (no./%)	68/18
Exact age, years (Mean ± SD)	69.2 ± 5.7
Educational level:	
primary school/vocational training (no./%)	63/17
secondary school (no./%)	162/44
university (no./%)	147/39
BMI, kg/m^2^ (Mean ± SD)	28.0 ± 5.0
pHDI-8 (Mean ± SD)	10.0 ± 4.3
nHDI-11 (Mean ± SD)	4.1 ± 2.2
MIND (Mean ± SD)	7.6 ± 1.6

**Table 2:** The correlation coefficients between selected psychological variables, dietary indexes, nutritional status, and other selected variables.

Nutritional Status and Psychological Symptoms	Age	BMI	Cigarettes per Day	pHDI	nHDI	MIND
Nutritional status (MNA)	−0.059	0.053	−0.067	0.035	0.080	−0.074
Distress (4DSQ)	0.101	0.003	0.069	0.064	0.062	−0.056
Depression (4DSQ)	0.112 *	0.081	0.034	0.050	0.119 *	−0.045
Anxiety (4DSQ)	0.007	0.085	0.135 *	0.023	0.092	−0.047
Somatisation (4DSQ)	0.125 *	0.130 *	0.076	0.038	0.059	−0.017
Depression (GDS)	0.064	0.060	0.097	0.001	0.092	−0.098
Perceived Stress (PSS-10)	−0.028	0.006	0.092	−0.031	0.056	−0.068

* *p* < 0.05.

**Table 3:** ANOVA results of the effect of gender on nutritional status and psychological symptoms (Mean ± SD).

Nutritional Status and Psychological Symptoms	Total	Gender		*p*
		F (*n* = 295)	M (*n* = 77)	
Nutritional status (MNA)	26.71 ± 2.06	26.61 ± 2.17	27.09 ± 1.54	0.066
Distress (4DSQ)	5.33 ± 4.29	5.83 ± 4.34	3.39 ± 3.49	<0.001 *
Depression (4DSQ)	0.37 ± 0.96	0.40 ± 0.98	0.23 ± 0.87	0.174
Anxiety (4DSQ)	0.85 ± 1.61	0.97 ± 1.73	0.39 ± 0.97	0.005 *
Somatisation (4DSQ)	5.77 ± 4.32	6.18 ± 4.40	4.22 ± 3.62	<0.001 *
Depression (GDS)	1.91 ± 2.21	2.05 ± 2.19	1.36 ± 2.21	0.015 *
Perceived Stress (PSS-10)	12.88 ± 6.88	13.50 ± 6.90	10.50 ± 6.10	<0.001 *

* *p* < 0.05.

**Table 4:** ANOVA results of the effect of levels of the non-healthy diet index on nutritional status and psychological symptoms (Mean ± SD).

Nutritional Status and Psychological Symptoms		nHDI		*p*
	Tertile 1	Tertile 2	Tertile 3	
Nutritional status (MNA)	26.54 ± 2.13	26.7 ± 1.86	26.92 ± 2.16	0.321
Distress (4DSQ)	4.74 ± 3.78	5.61 ± 4.36	5.62 ± 4.66	0.181
Depression (4DSQ)	0.15 ± 0.46 ^a^	0.41 ± 0.95 ^b^	0.54 ± 1.26 ^b^	0.006 *
Anxiety (4DSQ)	0.69 ± 1.47 ^a^	0.72 ± 1.46 ^a^	1.14 ± 1.87 ^b^	0.048 *
Somatisation (4DSQ)	5.26 ± 4.10	5.98 ± 4.32	6.06 ± 4.51	0.277
Depression (GDS)	1.55 ± 1.86 ^a^	1.82 ± 2.10 ^ab^	2.27 ± 2.57 ^b^	0.036 *
Perceived Stress (PSS-10)	12.09 ± 7.02	13.37 ± 7.09	13.16 ± 6.50	0.297

^a,b^ values with different letter inscriptions indicate statistically significant differences. * *p* < 0.05.

**Table 5:** ANOVA results of nutritional status (MNA) impact on psychological symptoms (Mean ± SD).

Psychological Symptoms	MNA		*p*
	Well (*n* = 339)	Risk (*n* = 33)	
Distress (4DSQ)	5.03 ± 4.10	8.33 ± 5.11	<0.001 *
Depression (4DSQ)	0.33 ± 0.92	0.73 ± 1.28	0.029 *
Anxiety (4DSQ)	0.77 ± 1.45	1.76 ± 2.68	0.000 *
Somatisation (4DSQ)	5.54 ± 4.13	8.12 ± 5.42	<0.001 *
Depression (GDS)	1.82 ± 2.18	2.76 ± 2.33	0.020 *
Perceived Stress (PSS-10)	12.51 ± 6.84	16.64 ± 6.16	<0.001 *

* *p* < 0.05.

**Table 6:** The adherence to the MIND and its relation to the examined features.

	High Adherence to MIND, 3rd Tertile			Middle or High Adherence to MIND, 2nd & 3rd Tertile			Low Adherence to MIND, 1st Tertile		
	*n*	Crude Model OR (CI95%), *p*	Adjusted Model ^1^ OR (CI95%), *p*	*n*	Crude Mode OR (CI95%), *p*	Adjusted Mode OR (CI95%), *p*	*n*	Crude Mode OR (CI95%), *p*	Adjusted Mode OR (CI95%), *p*
Mild or severe depression (GDS)	8	0.64 (0.28; 1.48), *p* = 0.296	0.70 (0.30; 1.64), *p* = 0.413	12	0.46 (0.21; 0.97), *p* = 0.041	0.47 (0.22; 1.00), *p* = 0.050	19	2.19 (1.03; 4.67), *p* = 0.041	2.13 (1.00; 4.56), *p* = 0.050
Moderate or high perceived stress (PSS10)	82	0.60 (0.39; 0.93), *p* = 0.022	0.60 (0.38; 0.93), *p* = 0.023	121	0.85 (0.56; 1.29), *p* = 0.451	1.15 (0.76; 1.75), *p* = 0.498	87	1.17 (0.77; 1.77), *p* = 0.451	1.15 (0.76; 1.75), *p* = 0.498
High perceived stress (PSS10)	16	0.58 (0.32; 1.08), *p* = 0.084	0.59 (0.32; 1.10), *p* = 0.098	33	0.79 (0.46; 1.35), *p* = 0.387	0.78 (0.45; 1.34), *p* = 0.370	31	1.27 (0.74; 2.18), *p* = 0.387	1.28 (0.74; 2.21), *p* = 0.370
Moderately elevated or high tendency to somatisation (4DSQ)	1	1.12 (0.61; 2.07), *p* = 0.717	1.10 (0.59; 2.06), *p* = 0.760	1	1.16 (0.64; 2.11), *p* = 0.624	1.22 (0.67; 2.24), *p* = 0.514	1	1.30 (0.08; 21.14), *p* = 0.853	0.98 (0.06; 16.97), *p* = 0.990
High score on depression (4DSQ)	1	0.95 (0.08; 10.67), *p* = 0.966	1.10 (0.10; 12.53), *p* = 0.938	1	0.38 (0.03; 4.27), *p* = 0.432	0.43 (0.04; 4.87), *p* = 0.493	2	2.63 (0.23; 29.48), *p* = 0.432	2.33 (0.21; 26.50), *p* = 0.493
Medium or high risk of depression (4DSQ)	6	0.95 (0.08; 10.67), *p* = 0.966	1.12 (0.61; 2.07), *p* = 0.717	7	0.76 (0.26; 2.22), *p* = 0.612	0.82 (0.28; 2.40), *p* = 0.710	7	1.32 (0.45; 3.85), *p* = 0.612	1.23 (0.42; 3.61), *p* = 0.710
Moderately elevated distress (4DSQ)	13	0.63 (0.32; 1.24), *p* = 0.181	0.69 (0.35; 1.37), *p* = 0.287	24	0.67 (0.37; 1.23), *p* = 0.197	0.71 (0.39; 1.29), *p* = 0.259	26	1.48 (0.81; 2.70), *p* = 0.197	1.42 (0.77; 2.60), *p* = 0.259
Risk of malnutrition (MNA)	16	0.52 (0.25; 1.08), *p* = 0.079	0.51 (0.25; 1.05) ^2^, *p* = 0.069	25	0.38 (1.17; 0.88), *p* = 0.023	0.37 (0.16; 0.85) ^2^, *p* = 0.019	8	2.60 (1.14; 5.95), *p* = 0.023	2.70 (1.17; 6.19) ^2^, *p* = 0.019

^1^ adjusted by age and BMI. ^2^ adjusted by age.

## Data Availability

The data presented in this study are available upon request from the corresponding author. The data are not publicly available due to privacy restrictions.

## Abbreviations

The following abbreviations are used in this manuscript:

MNA	The Mini Nutritional Assessment
FFQ	Food Frequency Questionnaire
4DSQ	The Four-Dimensional Symptom Questionnaire
GDS	Geriatric Depression Rating Scale
